# Toxicity of Titanium Dioxide–Cerium Oxide Nanocomposites to Zebrafish Embryos: A Preliminary Evaluation

**DOI:** 10.3390/toxics11120994

**Published:** 2023-12-06

**Authors:** Roberta Pecoraro, Elena Maria Scalisi, Stefania Indelicato, Martina Contino, Giuliana Coco, Ilenia Stancanelli, Fabiano Capparucci, Roberto Fiorenza, Maria Violetta Brundo

**Affiliations:** 1 Department of Biological, Geological and Environmental Science, University of Catania, Via Androne 81, 95124 Catania, Italy; elenamaria.scalisi@unict.it (E.M.S.); stefania.indelicato@phd.unict.it (S.I.); martina.contino@phd.unict.it (M.C.); giulicoco@gmail.com (G.C.); ilenia.stancanelli@hotmail.it (I.S.); mariavioletta.brundo@unict.it (M.V.B.); 2 Department of Chemical, Biological, Pharmaceutical and Environmental Science, University of Messina, Via F. Stagno D’Alcontres 31, 98166 Messina, Italy; fcapparucci@unime.it; 3 Department of Chemical Sciences, University of Catania, Viale A. Doria 6, 95125 Catania, Italy; rfiorenza@unict.it

**Keywords:** *Danio rerio*, ZFET, cardiotoxicity, engineered nanoparticles

## Abstract

The widespread use of metal nanoparticles in different fields has raised many doubts regarding their possible toxicity to living organisms and the accumulation and discharge of metals in fish species. Among these nanoparticles, titanium dioxide (TiO_2_) and cerium oxide (CeO_2_) nanoparticles have mainly been employed in photocatalysis and water depuration. The aim of this research was to evaluate the potential toxic effects, after a co-exposure of TiO_2_-3%CeO_2_ nanoparticles, on zebrafish development, using an acute toxicity test. Increasing concentrations of TiO_2_-3%CeO_2_ nanoparticles were used (0.1-1-10-20 mg/L). The heartbeat rate was assessed using Danioscope^TM^ software (version 1.2) (Noldus, Leesburg, VA, USA), and the responses to two biomarkers of exposure (Heat shock proteins-70 and Metallothioneins) were evaluated through immunofluorescence. Our results showed that the co-exposure to TiO_2_-3%CeO_2_ nanoparticles did not affect the embryos’ development compared to the control group; a significant difference (*p* < 0.05) at 48 hpf heartbeat for the 1, 10, and 20 mg/L groups was found compared to the unexposed group. A statistically significant response (*p* < 0.05) to Heat shock proteins-70 (Hsp70) was shown for the 0.1 and 1 mg/L groups, while no positivity was observed in all the exposed groups for Metallothioneins (MTs). These results suggest that TiO_2_-3%CeO_2_ nanocomposites do not induce developmental toxicity; instead, when considered separately, TiO_2_ and CeO_2_ NPs are harmful to zebrafish embryos, as previously shown.

## 1. Introduction

Engineered nanoparticles (ENPs) and nanomaterials (ENMs) of different sizes, shapes, and properties have been employed in several applications [1,2,3], although their daily consumption has raised some doubts about their possible effects on human and environmental safety [4,5]. Due to their small dimensions, ranging from 1 to 100 nm, ENPs have particular physical and chemical properties that are determined by their high surface area and nanoscale size [6]. Once ENPs are distributed into the environment, they interact with cells and organisms and can be adsorbed, causing potential risks. They are able to accumulate in cells and induce specific organ toxicity; therefore, there is a need to produce safer nanometric particles and especially to carry out toxicity tests that can be used for the development of rigorous guidelines [7].

Cerium dioxide nanoparticles (CeO_2_ NPs) have been used in fuel additives and polishing agents [8]. Globally, the estimated production of nano-CeO_2_ is around 1000 tons/year and it has become one of the most synthetized ENPs [9]. Upon its release into the aquatic environment, CeO_2_ may cause adverse effects (including alterations in the activity of brain enzymes, mortality, and behavioral responses, such as an alteration in the swimming speed of larvae, oxidative stress, genotoxicity, and the histological damage of target organs) on organisms at different trophic levels, such as *Carassius auratus* [10], *Artemia salina* [11,12], and freshwater fish [13,14].

Titanium dioxide nanoparticles (TiO_2_ NPs) are engineered nanoparticles that are very common worldwide since they have applications in many fields, such as paints, packaging, sunscreen, pharmaceuticals, water depuration, photoelectrochemical solar cells, and photovoltaic systems [15,16,17]. It is well known that titanium dioxide (TiO_2_) is a good photocatalyst that, under UV illumination, generates energy-rich electron–hole pairs that can be transferred to the surface of the TiO_2_. This promotes reactivity with the surface-absorbed molecules, leading to the production of active radicals [18]. The hydroxyl radicals (•OHs) and reactive oxygen species (ROS) on the TiO_2_ surface result in the oxidation of the polyunsaturated phospholipids of the cell membranes. In this regard, TiO_2_ has been used as an antimicrobial in packaging materials, where the eradication of microbes depends on the ROS that are released by the TiO_2_, leading to the oxidation of the bacterial cells and the cells’ death [19]. Moreover, the hydroxyl radicals cause the degradation of a wide variety of water pollutants [20,21]. For this reason, TiO_2_ has been greatly used in the area of wastewater treatment [22,23] and in the degradation of volatile organic compounds (VOCs) [24]. In sunscreen products, TiO_2_ is used as a UV filter to protect the skin from harmful UV rays when exposed to sunlight [25]. The ecotoxicological impact of TiO_2_ NPs has been noticed in different organisms, such as *Daphnia magna* [26], *Mytilus galloprovincialis* [27,28], *Artemia salina* [29], *Crassostrea gigas* [30], *Paracentrotus lividus* [31], and *Danio rerio* [32,33,34,35].

Therefore, the application of TiO_2_ and CeO_2_ NPs can increase the concentration of NPs in the environment with effects on the health of ecosystems and organisms.

Currently, the use of *Danio rerio* as a model to assess nanotoxicity is commonly accepted in the scientific research [36,37,38]. It is a small tropical fish, which offers several practical advantages concerning its manipulation, including its versatility, low cost of maintenance, and the ease of husbandry linked to its short life cycle [39,40]. The externally fertilized eggs develop rapidly, and its optical translucence allows the observation of the different developmental stages of embryogenesis, starting from the earliest stages [41]. At the same time, the embryos can be easily exposed to engineered nanomaterials; in particular, the Fish Embryo Toxicity (FET) test, based on zebrafish embryos, is an alternative approach to acute toxicity testing because it offers sensitivity and specificity as well as simplicity, economy, and quick execution [42]. The FET test has been successfully applied to a wide range of xenobiotics exhibiting diverse modes of action; it is also useful as an ecological risk assessment of the engineered nanoparticles, providing valuable information on their acute toxicity [43,44]. Several studies have used zebrafish embryos, because they are at a more sensitive life stage for the nanotoxicity assessment [38,45,46].

Moreover, the zebrafish is an organism with highly conserved organ systems and metabolic pathways that enable the evaluation of the toxicokinetics and toxicodynamics of xenobiotics, similar to mammalian models [47].

Nanoparticles can act on living cells at the nanometric level causing undesirable effects. In the literature, many studies have been performed to establish nanotoxicity using one single type of nanoparticle, while a combination of them has been poorly investigated. For this reason, the objective of this present work is to investigate the toxicity of nano-TiO_2_ and nano-CeO_2_ in a simultaneous exposure (co-exposure) to *Danio rerio* embryos. Once together, they can be considered a new nanocomposite. The endpoints included the survival rates and hatching, any malformations, an alteration in the cardiac function of embryos and larvae, and the biomarkers of exposure (Hsp70 and MTs).

## 2. Materials and Methods

### 2.1. Solutions Preparation

Powders of TiO_2_-3%CeO_2_ nanocomposites were supplied by the Department of Chemical Sciences (University of Catania). It is worth noting that this amount of CeO_2_ (3 wt%) was chosen because it was the most optimal for a good interaction with the TiO_2_. Because of the TiO_2_-3%CeO_2_ nanocomposite’ good photocatalytic performance and stability [48,49,50], we decided to test their toxicity.

A stock solution of TiO_2_-3%CeO_2_ nanocomposites in embryo medium was prepared. Then, based on our previous investigations using single nanoparticles (TiO_2_-NPs and CeO_2_-NPs), we chose the following concentrations to test: 0.1, 1, 10, and 20 mg/L. Using a sonicator equipped with a probe (SONOPULS, Bandelin electronic GmBH&Co, Germany), the stock solution was dispersed to avoid any natural aggregates of TiO_2_-CeO_2_. We carried out sonication for 2 min each, after which the stock solution was diluted in embryo medium, following Westerfield [51], to obtain working concentrations of 0.1, 1, 10, and 20 mg/L. Finally, the working solutions were sonicated.

### 2.2. Synthesis and Characterization of Nanocomposites

The wetness impregnation method was used on the as-prepared brookite [49] to obtain composites with the three weight percentages (wt%) of CeO_2_. Specifically, a solution of cerium nitrate hexahydrate was added for the impregnation of the brookite, and the obtained colloidal solution was dried at 120 °C and calcined at 350 °C for 4 h.

The TEM characterization was made with a Jeol 2100 F working at 200 kV. The dTEM (average particle size) was calculated by considering the number of particles (ni) of diameter (di), and this correlation of dTEM = Σ(nidi)/ni with around 150 metal oxides particles was considered for each sample [52].

**Figure 1:**
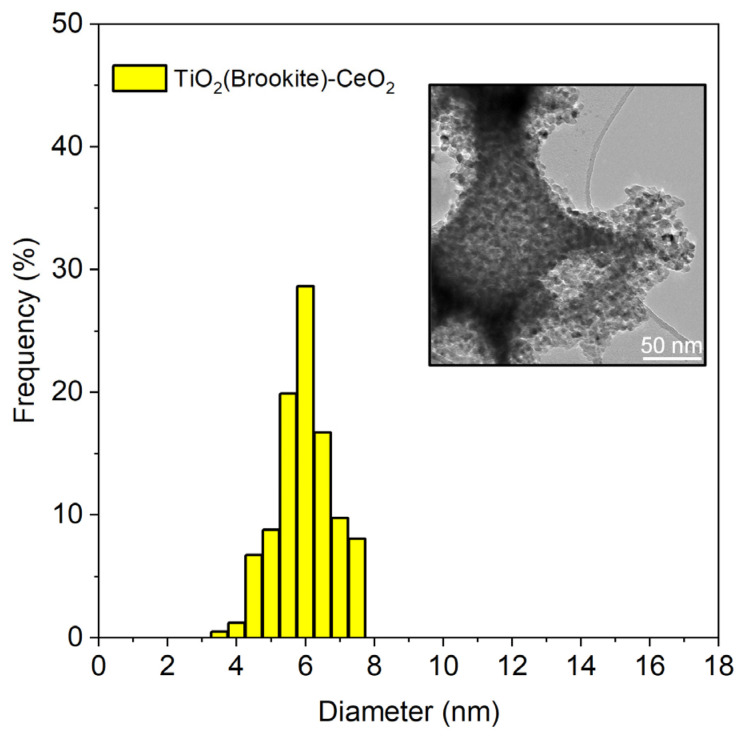
TEM image and particle size distribution of the TiO_2_-3%CeO_2_ sample.

A TEM image of the TiO_2_-3%CeO_2_ is presented in Figure 1. The average particle size was 6 nm.

### 2.3. Maintenance of Zebrafish and Embryo Collection

Adult *Danio rerio* (males and females) were raised in a fish room at the Fish Pathology and Experimental Centre of Sicily (CISS) of the Department of Veterinary Science (University of Messina). Wild-type adult zebrafish strains (4 months old) were used for egg production.

They were kept in a breeding room at 28.5 °C with a 10/14 h dark/light cycle, a water quality of pH 7.2 ± 0.3 and a 6.00 ppm dissolved oxygen content (DO). The night before the test, male and female fish (in a 2:1 ratio) were put into a hatching tank equipped with steel grids to avoid the predation of eggs by adults. The fish were left undisturbed overnight. The next morning, eggs were collected with Pasteur pipettes. All of the collected eggs were analyzed under a stereomicroscope (≥30-fold magnification) to discard infertile eggs and to select only eggs at the blastula stage. Then, the fertilized eggs were used for the acute toxicity test of the TiO_2_-3%CeO_2_.

### 2.4. Acute Toxicity Test of TiO_2_-3%CeO_2_ on Zebrafish Embryos

An acute toxicity test of the TiO_2_-3%CeO_2_ was performed according to the Organization for Economic Cooperation and Development (OECD) guideline 236 [42,53,54]. In total, 24 eggs at blastula stage were transferred into 24-well multi-plates, with one embryo per well, filled with 2 mL of solution. In each 24-well multi-plate, twenty embryos (one embryo per well) were exposed to TiO_2_-3%CeO_2_ concentration for assessment (0.1, 1, 10, and 20 mg/L), while four embryos were exposed to dilution water because they were internal plate controls (unexposed). We performed three replicates for each of the prepared multi-well plates. During the experiment, the multi-well plates were placed into the incubator (Heratherm Incubator, Thermo Scientific, Waltham, MA, USA) in order to maintain a constant temperature in the wells (26 ± 1 °C). The solutions in each well were replaced every 24 h (semi-static renewal) [53].

### 2.5. Toxicological Endpoints and DanioScope™ Analysis

The optical transparency of zebrafish embryos allowed the excellent visualization of their early life stages. They were observed up to 96 hpf using the E200 MV-R LED microscope (Nikon, Tokyo, Japan) equipped with a CMOS camera (Nikon). According to the OECD guidelines, we recorded four toxicological endpoints daily: coagulated embryos, lack of somite formation, non-detachment of the tail, and lack of heartbeat. A positive outcome in any one of these observations would indicate that the zebrafish embryo was dead.

The heartbeat in normal zebrafish development is visible after 48 h; videos and photos were recorded in order to upload them using the DanioScope^TM^ software (version 1.2) and to measure the heartbeat rate and body length.

On the video upload, we selected the heart area above the ventricle because the DanioScope^TM^ evaluates the changes in pixel density during ventricular contractions, giving a number of beats per minute (BPM). The uploaded images were also useful for the measurement of the body length of the larvae. After the calibration with a micrometer scale, in each larva a distance starting from the tail to the head was defined. The DanioScope^TM^ measured lengths automatically.

### 2.6. Immunohistochemical Analysis on Larvae

An immunohistochemical analysis was carried out to localize the Heat shock protein-70 (Hsp70) and metallothioneins (MTs) biomarkers in whole larvae (both in the control and exposed groups). Our standard protocol of immunohistochemical analysis that has been used for several experiments on zebrafish was followed [42].

At the end of exposure, all larvae (exposed and control groups) were fixed in 4% (*w*/*v*) formaldehyde for 20 min at room temperature. Subsequently, they were washed with PBS (pH 7.4, 0.1 M) and permeabilized with PBS-Triton X-100 (for 15 min) to improve the penetration of the antibody. The larvae were incubated with the primary antibodies anti-rabbit-Hsp70 (1:1000; GeneTex Irvine, CA, USA) and anti-mouse-MTs (1:1000; GeneTex Irvine, CA, USA). The incubation was performed in a humid chamber at 4 °C (overnight).

After the primary antibodies incubation was complete, the larvae were washed twice (each time for 5 min) in PBS-Tween 20 to remove the excess of primary antibodies. The larvae were incubated with the TRITC-conjugated anti-rabbit and FITC-conjugated anti-mouse secondary antibodies (1:1000) in the dark. Finally, the secondary antibodies were removed through washing in PBS-Tween 20 (2 times for 5 min) at room temperature, dehydrated in increasing alcohol solutions (70°, 80°, and 95°) for 1 min each, and air dried. The larvae were mounted with DAPI (Abcam, Cambridge, UK) and sealed with rubber cement. The images were captured with a NIKON DS-Qi2 camera connected to a fluorescence microscope. Secondary antibody TRITC-conjugated exhibits a red fluorescence, while secondary antibody FITC-conjugated exhibits a green fluorescence.

### 2.7. Statistical Analysis

Statistical analysis was performed through a one-way analysis of variance (ANOVA) test to compare differences between groups. This was followed by the Tukey test (*p* < 0.05), using the statistical software Past4Project (version Past4.03). Moreover, ImageJ software (version 1.53) was used to assess the positive area in each image obtained via the fluorescence microscope. Data are expressed as the mean ± SD.

## 3. Results

### 3.1. Toxicological Endpoints on Zebrafish Embryo

In order to analyze the toxic effect of TiO_2_-3%CeO_2_ nanocomposites on embryo development, the toxicological endpoints (the rate of coagulated embryos, hatching, and survival) at 24, 48, 72, and 96 hpf were measured. The coagulation of the embryos was the first endpoint evaluated. Coagulated embryos are milky white and appear dark under the stereomicroscope. At 24 hpf, the percentage of coagulated embryos was 1.38% for the 0.1, 10, and 20 mg/L groups, while for the control and 1 mg/L groups, no coagulated embryos were observed.

**Figure 2:**
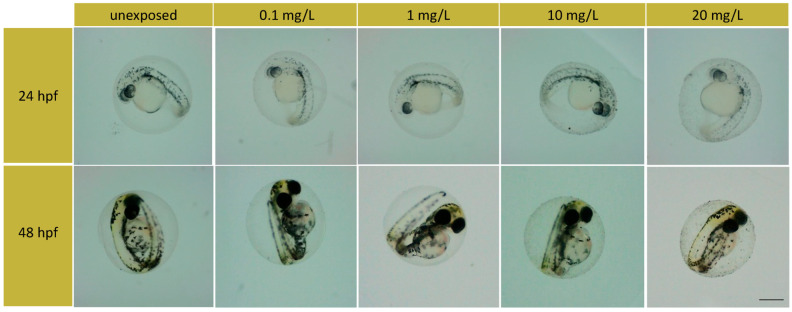
Phenotypes of embryos during exposure. Exposed groups and the unexposed group. Scale bar 400 µm.

The coagulation rate remained unchanged at 48 hpf, except for the 0.1, 10, and 20 mg/L exposed groups. All of these groups showed a percentage of coagulated embryos of 2.77%. These rates remained unchanged until the end of the test (at 96 hpf). From observations at 24 hpf and 48 hpf, no alterations in the embryo development were found. The embryo development was normal, as shown in Figure 2. Moreover, the survival rate was not altered by exposure until the end of the test. Only the 1 mg/L exposed group showed a survival rate of 100%, which was similar to the control group, while for the other exposed groups, the survival rate was 97.2%.

**Figure 3:**
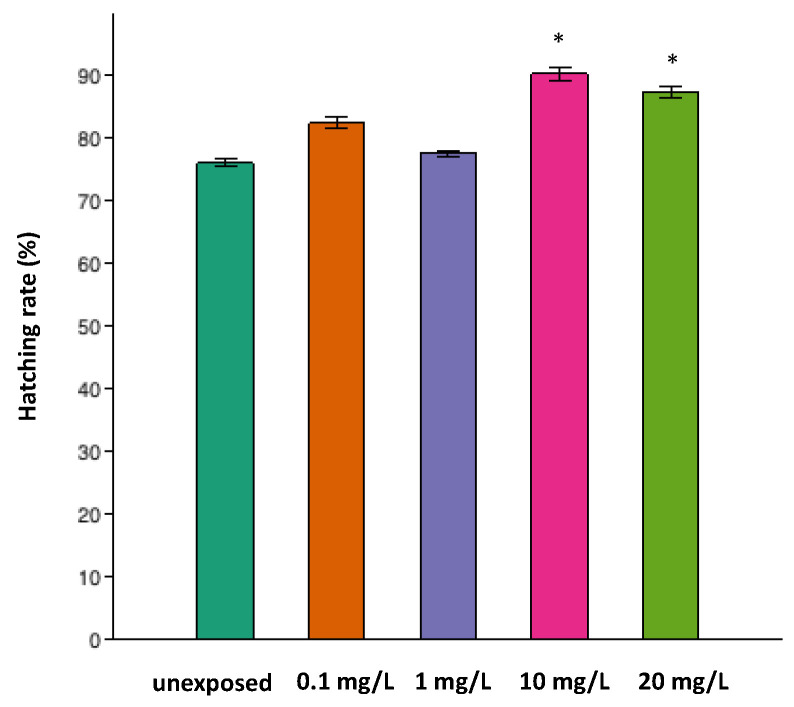
Hatching rate of embryos in the exposed groups and unexposed group. At 72 hpf, statistical significance was found for the groups exposed to 10 mg/L and 20 mg/L (*p* < 0.05). The asterisks (*) indicate a statistically significant difference in the one-way ANOVA followed by the Tukey test (*p* < 0.05) between the exposed groups and unexposed group at 72 h of exposure.

The hatching rates of the zebrafish embryos after the TiO_2_-3%CeO_2_ nanocomposites exposure are given in Figure 3. The hatching occurred between 72 and 96 hpf. At 72 hpf, the hatching rates of the 10 and 20 mg/L exposed groups were statistically higher than the unexposed group (*p* < 0.05).

The embryo developed inside the chorion, which acted as a protective barrier; during hatching, the loss of the chorion promoted the constant exposure of the larvae to the TiO_2_-3%CeO_2_ nanocomposites. However, in all the exposed groups, no morphological malformation compared to the unexposed group was observed.

**Figure 4:**
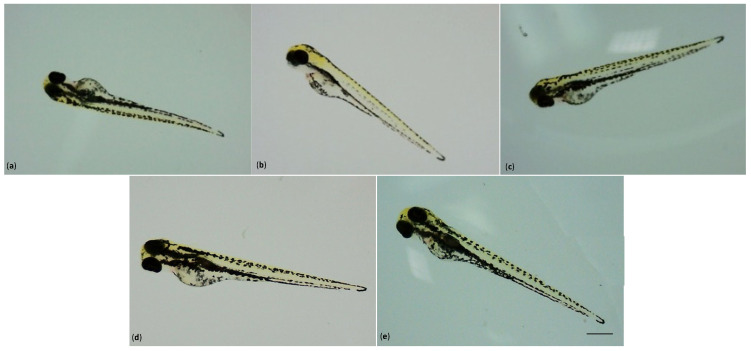
The 96 hpf phenotypes of larvae: (**a**) unexposed; (**b**) exposed to 0.1 mg/L; (**c**) exposed to 1 mg/L; (**d**) exposed to 10 mg/L; and (**e**) exposed to 20 mg/L of TiO_2_-3%CeO_2_. Scale bar 410 µm.

All larvae showed the complete development of a head, a notochord, a fin, pigmentation, a heart, and eyes (Figure 4).

### 3.2. Cardiology and Body Length Measurements Using Danioscope Software

In a normally developing zebrafish embryo, the heartbeat is visible after 48 h. Using the DanioScope^TM^ software (version1.2), the heartbeat rate was measured as beats per minute (BPM). At 48 hpf in the group exposed to 1 mg/L, 10 mg/L, and 20 mg/L of TiO_2_-3%CeO_2_, the heartbeat rates were statistically higher than those in the unexposed group (*p* < 0.05).

At 48 hpf, the additional structures that support the functions of the growing heart, such as the artery bulb, the valve cushions and leaflets, and the myocardial protuberances called trabeculae and epicardium, are usually missing [55]. However, the heart is located in the pericardial cavity and is clearly divided into a two-chambered heart by the constriction of the atrioventricular (AV) canal [56,57]. As a result, in its early form, the heart is more sensitive to developmental perturbations caused by exogenous substances. The pericardial edema of zebrafish due to nanoparticle exposure has been shown to be the common result of cardiotoxicity [58,59]. No statistical difference was observed in the heartbeat rates for all exposed groups at 72 hpf and 96 hpf. Regarding the body length of the larvae, no alterations were observed in the measurements for all exposed groups compared with the unexposed group.

### 3.3. Immunohistochemical Analysis

The immunohistochemical investigation for the Heat shock protein-70 (Hsp70) highlighted that exposure to the TiO_2_-3%CeO_2_ nanocomposites induced stress on the larvae. Using ImageJ software (version 1.53), a statistically significant difference (*p* < 0.05) in the Hsp70 biomarker between the unexposed group and the larvae exposed to 0.1 and 1 mg/L was observed. Between the exposed groups, the positivity was statistically higher for 1 mg/L compared to 10 mg/L and 20 mg/L (*p* < 0.05), whereas no statistically significant difference for 0.1 mg/L was observed.

**Figure 5:**
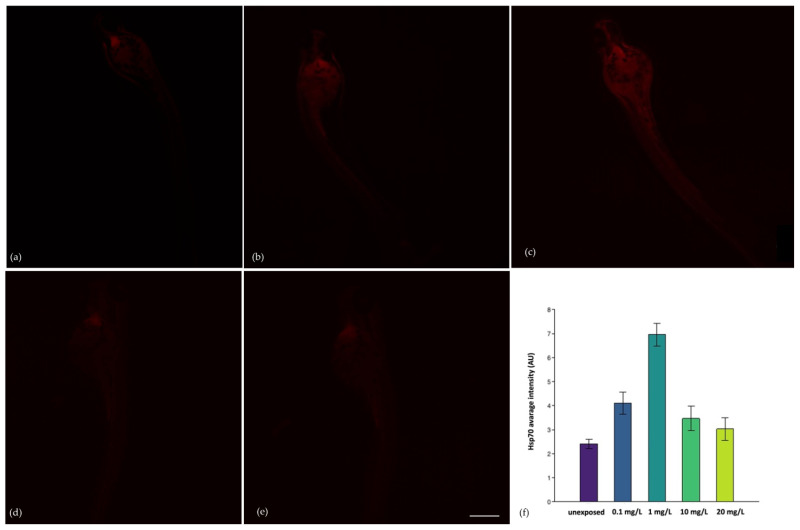
Hsp70 antibody staining at 96 hpf: (**a**) larva unexposed; (**b**) larva exposed to 0.1 mg/L of TiO_2_-CeO_2_ nanocomposites; (**c**) larva exposed to 1 mg/L of TiO_2_-CeO_2_ nanocomposites; (**d**) larva exposed to 10 mg/L of TiO_2_-CeO_2_ nanocomposites; and (**e**) larva exposed to 20 mg/L of TiO_2_-CeO_2_ nanocomposites. (**f**) The histograms represent the average intensity fluorescence (AU) of the biomarker Hsp70. Scale bar 100 µm.

Therefore, the lower concentrations of TiO_2_-3%CeO_2_ nanocomposites were able to induce environmental stress on the larvae. Figure 5 shows images of the larvae and their average fluorescence intensity (AU). Compared to the exposure of single TiO_2_ and CeO_2_ nanoparticles, the TiO_2_-3%CeO_2_ nanocomposites were unable to induce the expression of MTs, which are involved in protection against heavy metals and oxidative damage [60]. Consequently, no positivity was observed in all the exposed groups.

## 4. Discussion

Aquatic ecosystems are the final destination for several environmental contaminants, and, therefore, it is necessary to understand the potential harm of some new xenobiotics, such as engineered nanoparticles. The small size of ENPs has toxicological consequences, since they can enter cells and interfere with their functional status [61]. Zebrafish are increasingly employed as the animal model for studying the effects of ENMs due to the exceptional set of characteristics that they possess [62,63]. Their small size, which allows them to be easily housed, and their large number of spawned eggs are the most important features that make them an ideal candidate for short-term toxicity assays. The transparency of their eggs is also convenient for monitoring how compounds can alter physiological development.

In our study, we exposed zebrafish embryos in order to investigate the potential co-toxicity of nano-TiO_2_ and nano-CeO_2_ in a simultaneous exposure. The survival rates and hatching, any malformations, an alteration in the cardiac function of embryos and larvae, and the biomarkers of exposure expression (Hsp70 and MTs) were the endpoints evaluated.

Very low percentages of coagulated embryos at 24 hpf were found (1.38% for the 0.1, 10, and 20 mg/L exposed groups) compared to the control group and 1 mg/L group, where no coagulated embryos were observed. The percentages still remained unmodified at 48 hpf, except for the 0.1, 10, and 20 mg/L exposed groups (2.77%), whose rates remained unchanged until the end of the test (at 96 hpf).

We obtained the maximum survival rate for the 1 mg/L exposed group, which was similar to the control group (100%), while for the other exposed groups, the survival rate was 97.2%. Moreover, in all the exposed groups, no morphological malformation compared to the control group was observed, since the larvae showed normal development.

These results are aligned with the results of our previous study [64], where the toxicity of single CeO_2-_NPs to zebrafish embryos was evaluated by assessing different endpoints such as vitality, mortality, and the response to biomarkers of exposure (MTs, Hsp70, and EROD). After 96 h of exposure, no significant mortality was observed for both control groups and treated groups. Additionally, the control groups showed normal development where the hatching rates and survival rates were above 90%. After 96 h of exposure, there was no significant mortality and no sublethal effects such as hatching delay, heartbeat alteration, or malformation in embryonic development compared with the control group. The maximum mortality was 2% for the whole test period.

In a previous study [65], an early life stage test was performed with zebrafish embryos. The exposure (at 96 h) to nano-CeO_2_ concentrations (1, 10, and 100 mg/L) in the absence and presence of humic substances (HS) (10 and 40 mg C/L), as well as the HS alone (10 and 40 mg C/L), was assessed. The lethality, as well as sublethal developmental morphology and toxicological endpoints, was scored according to Hermsen et al. [66]. The embryos that were exposed to individual nano-CeO_2_ showed scoliosis, but the combination of nano-CeO_2_ and the higher concentration of HS (40 mg C/L) induced more toxicity, including pericardial oedema, yolk sac oedema, and scoliosis. Thus, the concomitant exposure of the HS and nano-CeO_2_ caused the most toxicity to the zebrafish embryos.

With regard to titanium dioxide nanoparticles (TiO_2_-NPs), it has been demonstrated that they cause early hatching and that prematurely hatched embryos have a smaller size and a larger yolk sac relative to body size [67]. In our study, the hatching rates of zebrafish embryos after the TiO_2_-3%CeO_2_ exposure occurred between 72 and 96 hpf. At 72 hpf, the hatching rates of the 10 and 20 mg/L exposed groups were statistically higher than those of the unexposed (*p* < 0.05).

In the present study, the heartbeat was measured as beats per minute (BPM) using DanioScope^TM^ software (version1.2), and we established that at 48 hpf, in the groups exposed to 1 mg/L, 10 mg/L, and 20 mg/L of TiO_2_-3%CeO_2_, the heartbeat rates were statistically higher than in the unexposed group (*p* < 0.05). No statistical difference was observed in the heartbeat rates for all exposed groups at 72 hpf and 96 hpf. In the study of Scalisi et al. [68], zebrafish embryos were exposed to TiO_2_-NPs (P25, Degussa, Sigma Aldrich) at different concentrations (1 mg/L, 2 mg/L, and 4 mg/L), showing an increase in BPM for the 1 mg/L group and a higher BPM for the 4 mg/L group. In a normally developing zebrafish embryo, the heartbeat is visible after 48 h, and the BPM values are physiologically around 120–180 bpm [69]. Moreover, no morphological alterations were found in the exposed groups compared with the unexposed one.

Regarding the body length of the larvae in our study, no changes were observed in the measurements for all exposed groups compared with the unexposed group. Scalisi et al. [68] demonstrated that the TiO_2_-NPs affected the body length of larvae because they exhibited a reduction compared to the unexposed group (*p* < 0.05) at 96 hpf. The mean body length in the 4 mg/L group was 172 μm, while in the unexposed group, it was 215 μm.

Another study [23] has taken into consideration the toxicity of molecularly imprinted TiO_2_ catalysts (synthesized using the sol–gel technique) to zebrafish eggs. The eggs were incubated with bare TiO_2_ and with TiO_2_ molecularly imprinted with Imidacloprid (TiO_2_ MI/Imid) for 96 hpf, demonstrating no toxicity to zebrafish embryos after exposure. With regard to the response of biomarkers, no expression of the anti-MTs antibody was detected in the whole body of larvae after treatment with the TiO_2_ and TiO_2_ MI/Imid. In our study, the exposure to TiO_2_-3%CeO_2_ nanocomposites did not induce the expression of any inducible MTs proteins.

The Hsp70 is a sensitive, stress-inducible member of the heat shock proteins family and is often expressed in a tissue-specific manner in a number of vertebrate species [70]. In the present study, a statistically significant difference (*p* < 0.05) in the Hsp70 biomarker between the unexposed group and the exposed larvae at the lowest concentrations (0.1 and 1 mg/L) was observed. The highest positivity was detected in the head of the larvae (as observed by other authors). It seems that an increase in the concentrations of NPs promotes aggregates, suggesting less interaction with the organism and fewer responses to physiological stress. In a study [71] considering the alteration in the immune system of zebrafish, which shows a lot of similarities with the immune system of vertebrates, some changes in gene expression in the liver tissue showed that there was an upregulation in Hsp70 genes after metal nanoparticles exposure. This aspect provides an explanation for the fact that metal nanoparticles exposure leads to immunotoxicity in zebrafish.

As described in a previous study [72], after metal nanoparticles exposure, some biomarker genes, such as MTs, which are considered to be a specific biomarker for metal toxicity, and also Hsp70, which is involved in the response to oxidative stress as well as general stressful conditions, were analyzed. This gene was overexpressed, with the highest-fold change in comparison to all the other genes tested in the research, demonstrating its involvement when engineered nanoparticles are present in the environment. Finally, in light of the results of the toxicological endpoints analyzed in this study, a co-exposure to TiO_2_-3%CeO_2_ nanocomposites does not affect zebrafish embryonic development. Therefore, it would be desirable to continue with the investigation and to plan long-term experimentation in order to have a broader overview of toxic effects

## 5. Conclusions

This study shows that a co-exposure of TiO_2_-3%CeO_2_ nanocomposites does not affect the development of zebrafish embryos. The association between TiO_2_ and CeO_2_ nanoparticles does not produce adverse effects on this species, while zebrafish larvae exposed to TiO_2_ or CeO_2_ nanoparticles alone show different results, such as alterations in the heart rate and the hatching rate, morphological changes, and the expression of biomarkers. On the contrary, the co-exposure did not show any outcomes that suggested an absence of toxicity in the concentrations that were tested. Currently, since there are no studies in the literature that evaluate the acute effects on zebrafish after a combined exposure to these two widespread engineered nanoparticles, it would be interesting to investigate the mechanisms of toxicity.

## Data Availability

Original data are available on request.
